# Sinonasal mucosal melanoma in The Netherlands between 2001 and 2021: a clinical and epidemiological overview of 320 cases

**DOI:** 10.1007/s00405-024-08717-7

**Published:** 2024-05-18

**Authors:** W. F. Julius Scheurleer, Lise J. van de Velde, Lot A. Devriese, Mischa de Ridder, Marieke W. J. Louwman, Gerben E. Breimer, Remco de Bree, Boukje A. C. van Dijk, Johannes A. Rijken

**Affiliations:** 1 Department of Head and Neck Surgical Oncology, University Medical Center Utrecht, Heidelberglaan 100, 3584 CX Utrecht, The Netherlands; 2 Department of Medical Oncology, University Medical Center Utrecht, Utrecht, The Netherlands; 3 Department of Radiation Oncology, University Medical Center Utrecht, Utrecht, The Netherlands; 4 Comprehensive Cancer Center The Netherlands (IKNL), Department of Research and Development, Utrecht, The Netherlands; 5 Department of Pathology, University Medical Center Utrecht, Utrecht, The Netherlands; 6 Department of Epidemiology, University Medical Center Groningen, Groningen, The Netherlands

**Keywords:** Mucosal melanoma, Paranasal sinus cancer, Nose cancer, Head and neck cancer, Sinonasal cancer

## Abstract

**Purpose:**

Sinonasal mucosal melanoma (SNMM) is a rare malignancy, characterised by high (local) recurrence rates and poor survival. Comprehensive understanding of tumour etiology is currently lacking, which complicates adequate tumour treatment. Besides examining trends in incidence, this study aims to assess the association between clinical characteristics, treatment practices and patient outcomes, with the objective of establishing a baseline from which SNMM management can be enhanced.

**Methods:**

All newly diagnosed SNMM cases in The Netherlands between 2001 and 2021 were included using data from The Netherlands Cancer Registry (NCR).

**Results:**

A total of 320 patients were included. The annual incidence rate for the overall population was stable over the inclusion period with an annual percentage change (APC) of only − 0.01%. The 5-year overall survival (OS) and relative survival (RS) were 24.5 and 32.4%, respectively. Relative survival did not increase over time. The addition of adjuvant radiotherapy to surgery was not associated with a higher OS and RS compared to surgery alone.

**Conclusion:**

Sinonasal mucosal melanoma is a rare disease with stable incidence rates in the Netherlands between 2001 and 2021. There has been no improvement in survival over the course of the inclusion period. The study reaffirms that adjuvant radiotherapy does not seem to improve patient outcomes. Given the generally poor outcomes for SNMM patients, novel therapeutic options ought to be considered in order to improve care.

## Introduction

Sinonasal mucosal melanoma (SNMM) is a rare type of cancer that arises from melanocytes in the mucosa of the nasal cavity and paranasal sinuses. The incidence of mucosal melanomas, including SNMM, is much lower than that of their cutaneous counterparts, constituting approximately 1% of all melanoma cases [1]. In the head and neck region, between 60 and 70% of mucosal melanomas originate in the nasal cavity and paranasal sinuses [2, 3]. Of these, the vast majority are situated in the nasal cavity, followed by the maxillary and ethmoid sinuses [4]. The pathogenesis of SNMM is still poorly understood, and no apparent risk factors have been identified to date. Moreover, these tumors are characterized by a distinct molecular profile and mutational signature that differs from cutaneous melanoma [5–8].

SNMM is characterized by high recurrence rates and poor survival [9–12]. Surgery is the primary treatment modality for SNMM, aiming to achieve clear surgical margins [4, 13–16]. This may be difficult to accomplish due to the complex anatomy of the nasal cavity and paranasal sinuses and the close relation to vital structures such as the orbit and the anterior skull base. Additionally, the occurrence of multifocal lesions in up to 25% of patients, possibly linked to extensive field cancerization, further complicates local tumor control [17]. Both open and endoscopic endonasal approaches (or a combination of both) are viable, depending on the extent of disease. However, an endoscopic approach is favored whenever feasible [14, 18]. Radiotherapy, administered following surgery, has been shown to improve local control but does not seem to improve survival [10, 13, 19, 20]. More recently, targeted therapy (e.g. BRAF-inhibitors) and immunotherapy (e.g. anti CTLA-4 and anti-PD-1 antibodies) have greatly influenced the management of (metastatic) cutaneous melanoma. Unfortunately, these treatments have not benefitted patients with mucosal melanoma to the same extent [21, 22]. SNMM survival hence remains unsatisfactory, necessitating the exploration of alternative approaches to manage relapse. The recently published UK guidelines for the management of head and neck mucosal melanoma recommend that rigorous follow-up, consisting of comprehensive clinical assessment and imaging during the first year after treatment could prove helpful in this matter, allowing for timely intervention in case of recurrence [23]. Following the first year, the frequency of follow-up may be gradually reduced. Although this approach has not yet been studied, the underlying reasoning warrants further exploration.

Due to the rarity of SNMM, most evidence regarding treatment selection is based on retrospective studies. Prospective research is difficult to conduct, adding to the importance of (inter)national databases for providing further insight. The present study aims to investigate the trends in the incidence of SNMM in the Netherlands and to identify which patient characteristics and treatment practices impact patient outcomes by using nationwide, prospective data. This research will point out factors associated with outcomes and areas for improvement, as well as successes in current treatment strategies.

## Methods

### Study population

Data was obtained through a structured query in The Netherlands Cancer Registry (NCR). The NCR is a nationwide database that has captured data on over 95% of newly diagnosed cancer patients in the Netherlands since 1989 [24, 25]. Dedicated registrars compile the registry by gathering data from patient health records based on a minimum dataset, which has expanded over time. All adult patients with a histopathologically confirmed SNMM diagnosed between January 1, 2001 and December 31, 2021 were included. Cases were excluded if an SNMM was discovered during autopsy, but the patient died due to other causes. Morphology and topography codes according to the International Classification of Diseases for Oncology (ICD-O-3) were used to identify cases in the NCR [26]. The topography codes encompassed the nasal cavity (C30.0), maxillary sinus (C31.0), ethmoid sinus (C31.1), frontal sinus (C31.2), sphenoid sinus (C31.3), paranasal sinus overlapping (C31.8), and paranasal sinus not otherwise specified (C31.9). The morphology codes covered malignant melanoma and its morphological subtypes (8720–8722, 8730, 8743, 8745, 8746, 8770–8772) [27].

### Operationalization

Tumors were staged according to the 7th edition (between 2010 and 2016) or the 8th edition (2017 onwards) of the Union for International Cancer Control (UICC) TNM-classification for mucosal melanoma of the upper aerodigestive tract [28, 29]. This classification was first introduced as part of the 7th edition. As a result, TNM-classification data was unavailable for patients diagnosed between 2001 and 2009. Information regarding the extent of disease was used to distinguish between local disease (T3-4bN0M0), regional disease (T3-4bN1M0), and distant metastases (T3-4bN0-1M1). The following variables were not available from the onset of the inclusion period because of the continuous expansion of the NCR: disease recurrence and progression were registered from 2017 onward; data regarding surgical margins were registered from 2018 onward. Information on radiotherapy of the neck was not available.

### Statistical analysis

The incidence was standardized to the Revised European Standard population and reported as the Revised European Standardized Rate (RESR) expressed per 100,000 [30]. As a result, these rates are comparable over the years despite changes in the size and age composition of the Dutch population. Joinpoint Trend Analysis Software version 4.2.0.2 was used to calculate trends in the standardized incidence rates and reported as an annual percentage change (APC) with 95% confidence intervals (95% CI). Stata/SE version 17.0 was used for all other analyses. Q-Q plots were used to assess normality of data. Patient characteristics were reported as means and standard deviations (SD) or medians with the 25th and 75th percentile (p25-p75) for normally and non-normally distributed variables, respectively. Overall survival (OS) was measured from the date of diagnosis until linkage to the municipal registry to obtain vital status and was calculated using the Kaplan–Meier estimator. The Cox proportional hazards model was used for univariable and multivariable analyses. The proportional hazards assumption was visually tested for categorical variables, and interaction with time was tested for continuous variables. The assumption was met for all variables. The relative survival (RS) rate was defined as the ratio of observed survival in the study population to the expected survival rate in the general Dutch population (obtained from Statistics Netherlands) by age, sex, and year. The relative excess risk (RER) was calculated in a univariable and multivariable model. Age at the time of diagnosis and sex were included in the multivariable analysis regardless of statistical significance. Other variables with a probability (*P*) value < 0.10 in the univariable analysis were introduced in the multivariable analysis and eliminated in a stepwise-backward fashion. Probability values < 0.05 were considered statistically significant for all tests. The variables multifocality of disease and resection margins were excluded from the analysis due to insufficient data. Calculating disease-free survival was not possible due to the lack of registration of recurrences prior to 2017. In order to ensure an adequate sample size per variable category, “surgery + radiotherapy” and “surgery + systemic (+ radiotherapy)” were grouped for the OS Cox proportional hazard analysis and RER analysis.

## Results

### Trends in incidence

**Fig. 1:**
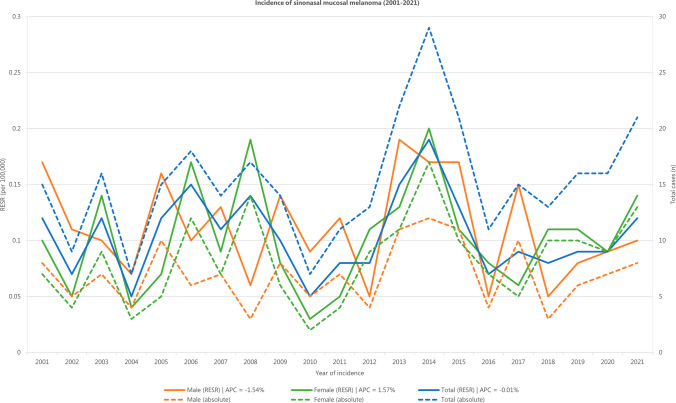
Incidence rates of sinonasal mucosal melanoma in the Netherlands between 2001 and 2021. The solid lines represent the annual incidence expressed as RESR. The dashed lines indicate the absolute incidence. *RESR* revised European standardized incidence rate, *APC* annual percentage change

The RESR and absolute incidence over time in The Netherlands between 2001 and 2021 are displayed in Fig. 1. The number of yearly diagnosed cases ranged from seven in 2004 and 2010 to 29 in 2014. The annual incidence rate for the entire population was stable with an APC of − 0.01% (95% CI − 2.50 to 2.60%. There appeared to be a slight downward trend for male patients (APC =  − 1.54%, 95% CI − 4.70 to 1.70%) and a slight upward trend for female patients (APC = 1.57%, 95% CI − 2.30 to 5.60%), but neither was statistically significant.

### Study population

The studied cohort consisted of 320 patients with a primary mucosal melanoma of the nasal cavity and paranasal sinuses, diagnosed in the Netherlands between 2001 and 2021. The clinical characteristics are shown in Table 1.

**Table 1:** Patient characteristics for patients (n = 320) with sinonasal mucosal melanoma diagnosed between 2001 and 2021 in The Netherlands

Age at diagnosis	Median	p25-p75
Years	73	65–81.5
Sex	N	%
Male	146	45.6
Female	174	54.4
Primary tumor site	N	%
Nasal cavity	262	81.9
Maxillary sinus	32	10.0
Ethmoid sinus	14	4.4
Sphenoid sinus	3	0.9
Frontal sinus	2	0.6
Sinonasal NOS	7	2.2
cT classification^a^	N	%
T3	95	48.7
T4a	54	27.7
T4b	26	13.3
Unknown	20	10.3
cN classification	N	%
N0	248	77.5
N1	30	9.4
Unknown	42	13.1
cM classification	N	%
M0	287	89.7
M1	33	10.3
Clinical stage (simplified)	**N**	**%**
Local disease	237	74.1
Regional disease	24	7.5
Distant metastases	33	10.3
Unknown	26	8.1
Primary tumor treatment modality	N	%
Surgery	63	19.7
Surgery + radiotherapy	176	55.0
Surgery + systemic (+ radiotherapy)^b^	6	1.9
Radiotherapy	32	10.0
Systemic (+ radiotherapy)^c^	9	2.8
None/BSC	34	10.6
Follow-up	Median	p25-p75
Median duration (months)	20.2	8.3–43.5
Outcome	N	%
Alive	70	21.9
Deceased	250	78.1

*p25-p75* 25th and 75th percentile; *NOS* not otherwise specified

^a^Only pertains to cases diagnosed between 2010 and 2021

^b^Includes patients treated with surgery followed by either (radiotherapy with subsequent) chemotherapy or immune(radio)therapy

^c^Includes patients treated with either (radiotherapy with subsequent) chemotherapy/targeted therapy, or immune(radio)therapy

Out of 320 patients, a slight majority (54.5%) was female. The vast majority of patients (81.9%) presented with a tumor of the nasal cavity. Two hundred thirty-seven patients (74.1%) were diagnosed with local disease, 24 (7.5%) had cervical lymph node metastases, and 33 (10.3%) had distant metastases upon diagnosis. For 26 patients (8.1%), the clinical stage was unknown. A total of 245 patients (76.6%) underwent surgery, with or without adjuvant treatment, 32 (10.0%) received radiotherapy only, and 34 (10.6%) received best supportive care.

### Outcome

**Fig. 2:**
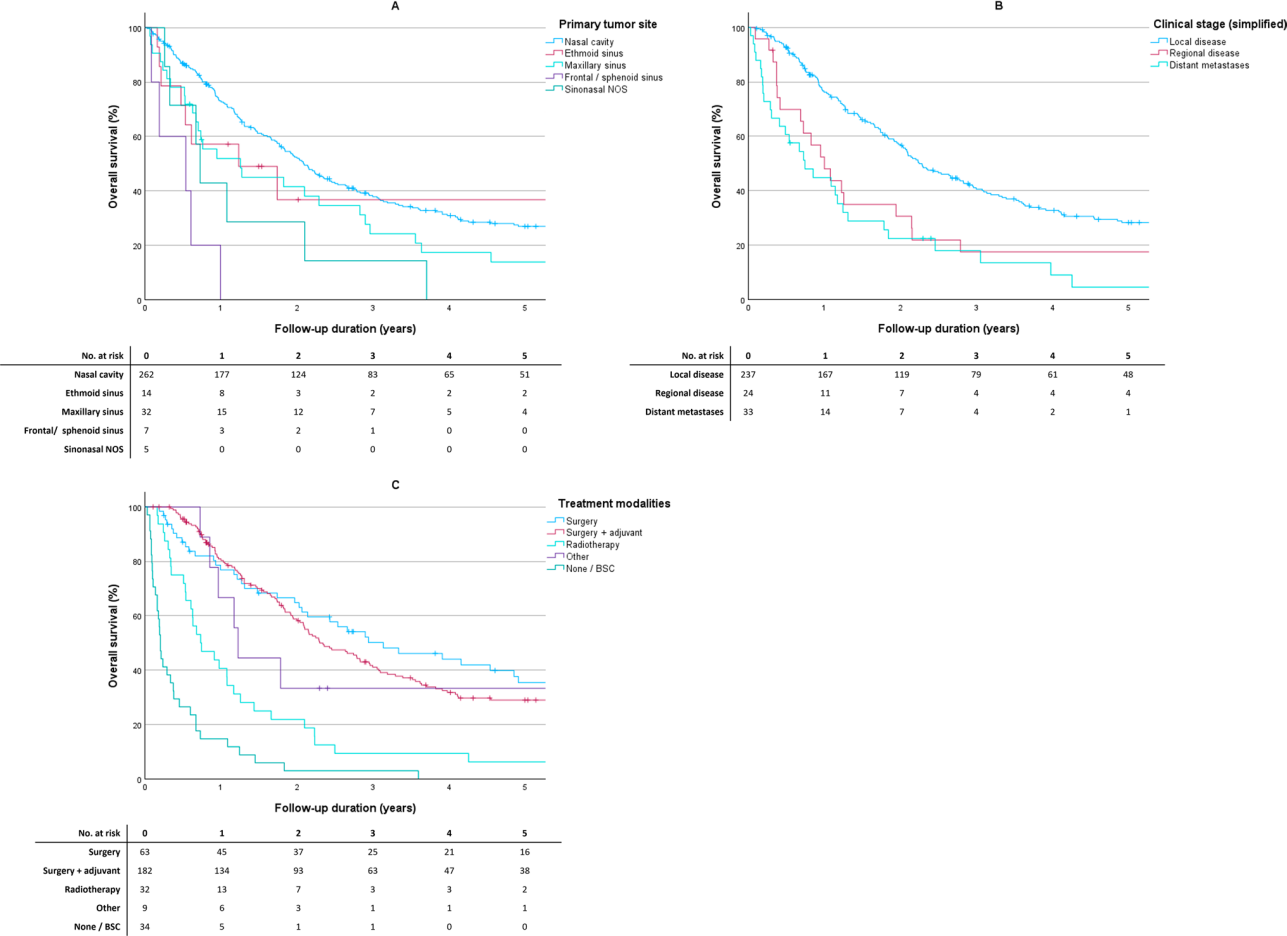
Kaplan–Meier survival estimates for overall survival in sinonasal mucosal melanoma in the Netherlands between 2001 and 2021. A is primary tumor site, B is clinical stage (simplified); C is treatment modality. *BSC* best supportive care

The median duration of follow-up was 20.2 months (p25-p75, 8.3–43.5). At the time of linkage to the municipal registry, 70 patients (21.9%) were alive and 250 (78.1%) had died. The 1-, 2-, 3-, and 5-year OS for the entire study population were 67.7% (95% CI 62.2–72.6%), 49.0% (95% CI 43.2–54.5%), 34.6% (95% CI, 29.2–40.1%), and 24.5% (95% CI 19.6–29.7%), respectively. The Kaplan–Meier survival estimates for OS, stratified by primary tumor site, clinical stage (simplified), and treatment modality, are shown in Fig. 2.

The findings of the Cox proportional hazard analysis are shown in Table 2. A statistically significant association was observed with age at the time of diagnosis, primary tumor site, clinical stage, and treatment modality in univariable analysis. This association remained significant for the aforementioned variables in multivariable analysis. Notably, there was no difference in the hazard of dying between patients who underwent surgery and adjuvant treatment, and patients who underwent surgery alone after adjustment for age, tumors site and clinical stage (HR = 1.16, 95% CI 0.80–1.68).

**Table 2:** Univariable and multivariable Cox proportional hazard analysis for overall survival

	Univariable			Multivariable^a^		
	HR	95% CI	*P*-value	HR	95% CI	*P*-value
Age at time of diagnosis (per 10 years)	1.40	1.24–1.57	** < 0.001**	1.29	1.13–1.47	** < 0.001**
Sex			0.258			
Male	ref	n.a				
Female	0.87	0.68–1.11				
Primary tumor site			** < 0.001**			** < 0.001**
Nasal cavity	ref	n.a		ref	n.a	
Maxillary sinus	1.57	1.06–2.34		1.54	0.98–2.42	
Ethmoid sinus	1.08	0.53–2.19		1.07	0.48–2.36	
Frontal/sphenoid sinus	6.64	2.68–16.5		6.34	2.40–16.7	
Clinical stage simplified			** < 0.001**			**0.003**
Local disease	ref	n.a		ref	n.a	
Regional disease	1.70	1.09–2.66		1.78	1.08–2.91	
Distant metastases	2.70	1.82–4.00		2.03	1.25–3.29	
Treatment modality			** < 0.001**			** < 0.001**
Surgery	ref	n.a		ref	n.a	
Surgery + adjuvant therapy^b^	1.23	0.87–1.74		1.16	0.80–1.68	
Radiotherapy	3.07	1.93–4.90		1.76	1.03–3.02	
Systemic (+ radiotherapy)^c^	1.87	0.84–4.18		0.91	0.34–2.44	
None/BSC	10.5	6.51–16.8		4.59	2.54–8.31	

Statistically significant *P*-values are depicted in bold

Age was included as a continuous variable per 10 years

*HR* hazard ratio, *95% CI* 95% confidence interval, *BSC* best supportive care, *n*.*a*. not applicable

^a^Variables with a *P*-value < 0.10 in univariable analysis were included in multivariable analysis. Sex was subsequently excluded in a stepwise backward manner

^b^Includes both patients who received either surgery + radiotherapy or surgery + systemic (+ radiotherapy)

^c^Includes patients treated with (radiotherapy with subsequent) chemotherapy, immune(radio)therapy, targeted therapy, or targeted therapy followed by radiotherapy

The 1-, 2-, 3-, and 5-year RS for the entire cohort were 76.1% (95% CI 70.9–80.5%), 59.6% (95% CI 53.6–65.0%), 43.7% (95% CI 37.1–50.1%), and 32.4% (95% CI 25.7–39.3%), respectively. RS was lower for patients with a tumor of the maxillary sinus compared to those with a tumor of the nasal cavity, regardless of the interval. Additional subgroup analyses for RS could not be performed due to low numbers.

**Fig. 3:**
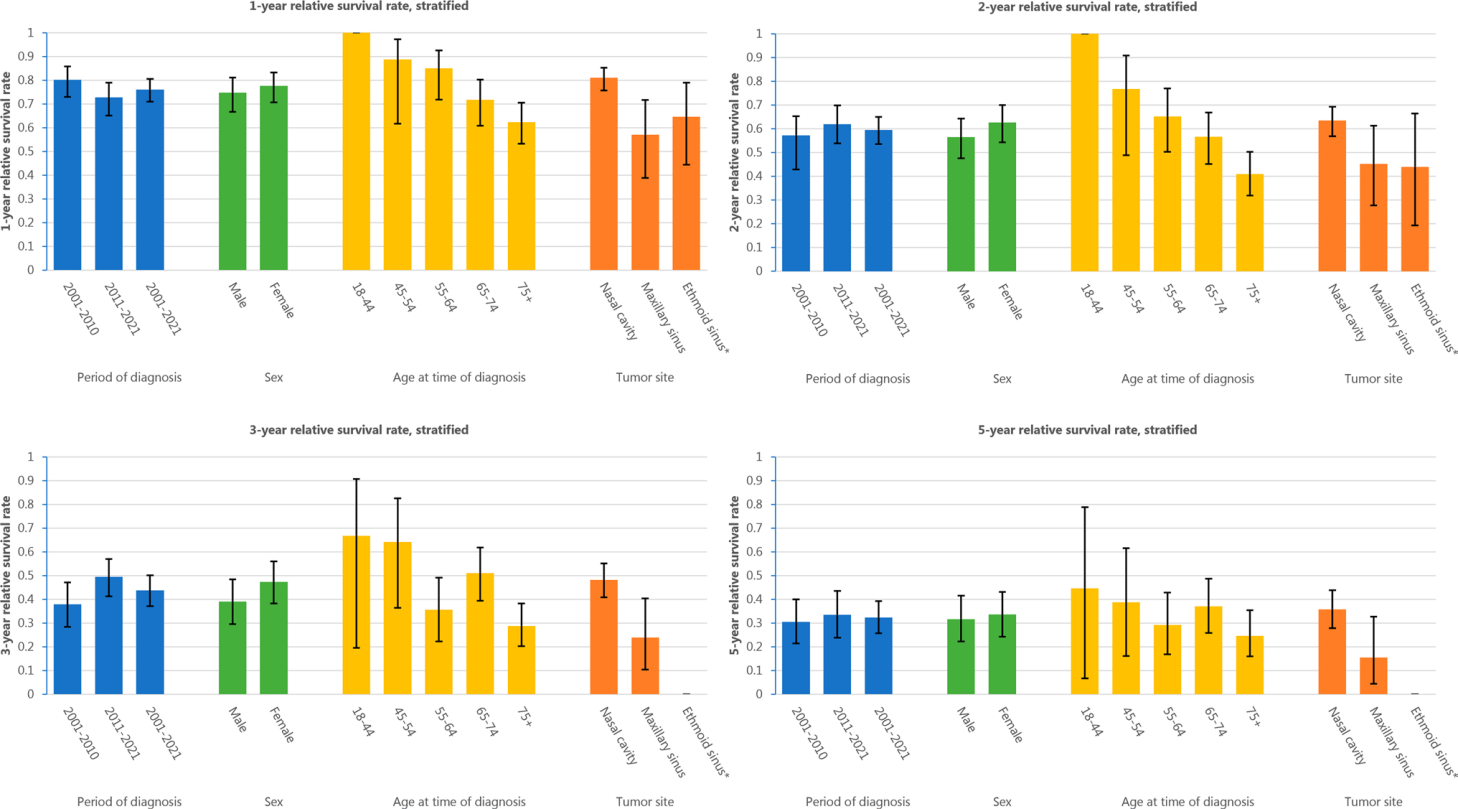
Relative survival rates at 1, 2, 3, and 5 years for sinonasal mucosal melanoma in the Netherlands between 2001 and 2021, stratified by period of diagnosis, sex, age at time of diagnosis, and tumor site. *3-year and 5-year RS rate for ethmoid sinus patients could not be calculated due to low numbers

The RER was statistically significant for age, primary tumor site, clinical disease stage, and treatment modality in the univariable analysis (Table 3). This association persisted for all variables except age in a multivariable model. Patients who underwent surgery and adjuvant treatment appeared to have a higher RER (RER = 1.28, 95% CI 0.81–2.02) than patients who underwent surgery alone after adjusting for covariates, although this was not statistically significant (see Fig. 3).

**Table 3:** Univariable and multivariable relative excess risk (RER) analysis

	Univariable			Multivariable^a^		
	RER	95% CI	*P*-value	RER	95% CI	*P*-value
Age at time of diagnosis (per 10 years)	1.29	1.13–1.48	** < 0.001**			
Sex			0.319			
Male	ref					
Female	0.86	0.64–1.16				
Primary tumor site			** < 0.001**			**0.018**
Nasal cavity	ref			ref		
Maxillary sinus	1.92	1.25–2.95		1.60	0.97–2.65	
Ethmoid sinus	1.29	0.60–2.78		1.12	0.47–2.66	
Frontal/sphenoid sinus	9.89	4.03–24.3		3.83	1.44–10.1	
Clinical stage simplified			** < 0.001**			**0.005**
Local disease	ref			ref		
Regional disease	1.80	1.09–2.98		1.57	0.88–2.82	
Distant metastases	3.57	2.35–5.43		2.46	1.40–4.32	
Treatment modality			** < 0.001**			** < 0.001**
Surgery	ref			ref		
Surgery + adjuvant therapy^b^	1.44	0.92–2.26		1.28	0.81–2.02	
Radiotherapy	3.81	2.15–6.74		2.04	1.04–4.02	
Other	2.64	1.08–6.42		0.73	0.22–2.43	
None/BSC	14.3	8.38–24.5		6.95	3.56–13.6	

Statistically significant *P*-values are depicted in bold

Age was included as a continuous variable per 10 years

*RER* relative excess risk, *95% CI* 95% confidence interval, *BSC* best supportive care

^a^Variables with a *P*-value < 0.10 in univariable analysis were included in multivariable analysis. Age at time of diagnosis and sex were subsequently excluded in a stepwise backward manner

^b^Includes both patients who received either surgery + radiotherapy or surgery + systemic therapy (+ radiotherapy)

## Discussion

This study underlines the rarity of SNMM in the Netherlands. The incidence rate was stable over the inclusion period. Long-term OS and RS for patients is generally poor and has not significantly improved over time. Moreover, the addition of adjuvant treatment modalities to surgery did not seem to improve outcomes for patients compared to surgery alone.

The APC over standardized incidence rates showed no change over the inclusion period. Previous studies have contrastingly reported increased rates over time. Jangard et al*.* have reported an upward trend in in Sweden between 1960 and 2000 [31]. A similar upward trend has been reported across Europe and the United States between 2000 and 2007[32]. Because our understanding of the etiology of SNMM is limited, it is unclear what this difference may be attributed to.

Survival rates for patients in this study were poor, with a 5-year OS of 24.5%, which is in line with previous literature: reported 5-year OS rates varied between 22 and 28% [10–12, 33]. This poor survival of patients is reflected in the UICC TNM-classification for mucosal melanoma of the upper respiratory tract in which T-stage starts at T3. However, this staging system has been shown to insufficiently correlate with prognosis [34, 35]. Clinically manifested lymph node metastases were found in 9.4% of patients, slightly exceeding the proportion of clinical regional disease (7–8%) reported by previous studies [11, 31, 33]. Information on (elective) treatment of the neck was not available for this cohort. Elective neck treatment is not commonplace because of the infrequent occurrence of occult nodal metastases in cN0 SNMM patients [11, 23]. Sentinel lymph node biopsy has been widely adopted and incorporated into standard care for cutaneous melanoma. However, there is limited evidence supporting this procedure in SNMM [36, 37]. The majority of patients in this cohort were treated with surgery and adjuvant radiotherapy. Notably, these patients had worse OS and RS compared to those who underwent surgery alone. Although adjuvant radiotherapy has been shown to improve local control, it does not appear to influence OS [8, 10, 19, 38, 39]. Outcomes for those who received radiotherapy alone were significantly worse. This was most likely due to bias by indication, given that single modality radiotherapy is typically reserved for palliative patients or those unfit for surgery. Primary radiotherapy appears to have little effect on outcomes [33, 39, 40].

Only eight patients had been treated with systemic therapy, being either targeted therapy (i.e. BRAF-inhibitors) or immunotherapy (ipilimumab, nivolumab, or pembrolizumab). As such, the influence of these treatment modalities on survival could not be adequately assessed. Targeted therapy has shown excellent results in metastasized cutaneous melanoma as BRAF-mutations are present in more than half of all patients [41]. In contrast, BRAF-mutations have been described in less than 10% of SNMM cases [8, 42–44]. Although the introduction of immunotherapy has greatly influenced care for patients with cutaneous melanoma, there has not been a similar improvement in survival for SNMM patients [21, 22]. There is a clear need for identification of new therapeutic targets and beneficial management strategies for SNMM. The role of immunotherapy, in the adjuvant setting, either with or without radiotherapy as well as the efficacy of several novel compounds, are currently under investigation [45] .

The present study cohort was assembled using the NCR where trained data registrars, ensuring meticulous data accuracy, collect data from the patient’s health record 5–9 months after diagnosis. It is imperative, however, to acknowledge several limitations. The extraction of information by registrars was contingent upon its recording in a patient's health record, thereby underscoring the significance of consistent and detailed documentation practices across individual hospitals. Information regarding treatment intent was not available and as such the distinction between curative and palliative treatment could not be made. Furthermore, cause of deaths was not available so cancer-specific death could not be analyzed. However, relative survival provided a close approximation of disease-specific survival. Similarly, data on disease progression or recurrence would have been helpful to be able to better understand the path from diagnosis to death. Unfortunately, this information was not available in sufficient detail at the time of this study. However, future reports are expected to be able to provide further insight as the collection of these variables has started in 2017.

## Conclusion

Sinonasal mucosal melanoma is a rare disease with stable incidence rates in The Netherlands between 2001 and 2021. There has been no improvement in survival over the course of the inclusion period. Moreover, adjuvant radiotherapy does not seem to improve patient outcomes. Given the generally poor outcomes for SNMM patients, novel therapeutic strategies ought to be considered in order to improve care.

## Data Availability

The authors are not at liberty to share data provided by the Netherlands Comprehensive Cancer Organization.
